# ﻿Karyological analysis of *Acanthocephalusranae* (Echinorhynchida): expanding the cytogenetic knowledge in acanthocephalans

**DOI:** 10.3897/zookeys.1243.153591

**Published:** 2025-06-25

**Authors:** Martina Orosová, Anna Marková

**Affiliations:** 1 Institute of Parasitology, Slovak Academy of Sciences, Hlinkova 3, 040 01 Košice, Slovakia Institute of Parasitology, Slovak Academy of Sciences Košice Slovakia; 2 Department of Zoology, Faculty of Natural Sciences, Comenius University, Ilkovičova 6, 842 15 Bratislava, Slovakia Comenius University Bratislava Slovakia

**Keywords:** Acanthocephala, chromosome, differential staining, fluorescence in situ hybridization, histone H3, karyotype, ribosomal genes

## Abstract

This study presents the first comprehensive cytogenetic analysis of the common amphibian parasite *Acanthocephalusranae*. A combination of classical cytogenetic methods and molecular techniques, including the fluorescence in situ hybridization (FISH) mapping of ribosomal and histone H3, was conducted. The karyotype consisted of three metacentric autosomes and either one submetacentric X chromosome in males or two submetacentric X chromosomes in females, resulting in a chromosome number of 2n = 7/8 (male/female). FISH mapping revealed that the ribosomal genes are located separately, with 18S rDNA situated on the X chromosomes and 5S rDNA on chromosome pair No. 3. The hybridization signals of histone H3 genes were dispersed across all chromosomes without any discernible pattern. Additionally, differential staining identified GC-rich heterochromatin at the ends of all chromosomes. These findings significantly expand the limited karyotypic data available for acanthocephalan parasites, of which the karyotype and/or chromosome number is known for only 1% of described species, and molecular cytogenetic techniques have been applied in just four species. The karyotype characteristics of *A.ranae* were also compared with other cytogenetically described thorny-headed worms within the order Echinorhynchida.

## ﻿Introduction

Acanthocephala represents a small group of dioecious endoparasites with complex life cycles involving arthropods as intermediate hosts and vertebrates as definitive hosts (Perrot-Minnot et al. 2023). Despite advancements in modern omics approaches, an increasing number of molecular phylogenies (Gao et al. 2022; Ru et al. 2022), and the inclusion of new molecular markers, the phylogenetic relationships and taxonomy within this group remain unresolved (Gazi et al. 2016), leaving many questions unanswered. Cytogenetics provides a valuable tool for addressing these issues, as chromosomes are fundamental hereditary elements of the genome. Insights into karyological characteristics can illuminate evolutionary relationships that may not be evident through morphological or molecular analyses alone (Dobigny et al. 2004). Additionally, information on genome organization, genome size, ploidy levels, and the chromosomal rearrangements involved in species speciation cannot be inferred solely from sequencing data (Ruiz-Herrera et al. 2012; Deakin et al. 2019).

There are two fundamental cytogenetic approaches for studying chromosomes in the karyotype: classical and molecular techniques. In Acanthocephala, most existing knowledge derives from classical cytogenetic techniques, with Giemsa staining-based karyotyping historically being the primary method employed. To date, basic chromosome data, such as chromosome number and morphology, have been documented for only 13 of the 1,270 described species. In these studies, the diploid chromosome number is typically reported as 2n = 7/8 (male/female) (Orosová et al. 2023: suppl. table S1). The development of molecular techniques, including molecular karyotyping and fluorescence in situ hybridization (FISH), has significantly advanced the study of karyotype structures. These approaches enable precise identification of chromosome homologues and investigation of chromosomal rearrangements and their evolutionary origins. Molecular methods are often integrated with classical cytogenetic techniques, creating a synergistic effect that enhances the resolution and scope of evolutionary cytogenetic studies (Orosová et al. 2021; Gouvi et al. 2022; Cabral-de-Mello et al. 2023; Mora et al. 2023). Among these, FISH is particularly prominent, as it facilitates the mapping of specific DNA sequences on chromosomes, making it a cornerstone method in evolutionary cytogenetics. Highly repetitive, tandemly arranged sequences, such as ribosomal and histone genes, have proven to be effective cytogenetic markers. Ribosomal genes are divided into two families: the major rRNA (18S, 5.8S, and 28S rRNA) and the minor rRNA (5S rDNA). These genes are typically organized in clusters of tandemly repeating units ranging from a few to thousands of copies (Long and Dawid 1980; Dalíková et al. 2023). Due to their universal occurrence in eukaryotic genomes and the flexible number, position and structure of their loci, ribosomal genes are considered key features at the species, genus, or group level. This makes them important markers for karyotypic and phylogenetic studies (Cabral-de-Mello et al. 2010; Garcia et al. 2012; Sochorová et al. 2021). Despite their utility, ribosomal genes exhibit highly dynamic evolution, and changes in their genomic distribution do not always correspond to chromosomal rearrangements (Poletto et al. 2010). In contrast, histone genes are conserved both in their protein sequences and in their genomic distribution, which makes them reliable chromosomal markers for the detection of chromosomal rearrangements. In acanthocephalans, molecular cytogenetics has been applied to only four species (Bombarová et al. 2007; Orosová et al. 2023; Marková et al. 2024; Orosová and Marková 2024). Mapping of 18S rDNA and/or histone H3 genes has revealed distinct, species-specific distribution patterns in all investigated species. Additionally, FISH-based telomere analysis in various platyhelminths and acanthocephalans demonstrated that none of the known telomeric motifs are present on the chromosomes of *Pomphorhynchuslaevis* (Bombarová et al. 2009). This finding suggests the presence of an unidentified telomeric motif or the loss of telomeric repeats, potentially replaced by alternative telomere maintenance mechanisms.

To expand our understanding of karyology within this group of parasites, we re-examined the karyotype of *Acanthocephalusranae*, a widespread endoparasite of anurans in Europe and Asia. Previous cytogenetic data for this species are limited to basic chromosome number and morphology, based on studies conducted over 60 years ago (Hamann 1891; John 1957). One early study proposed a mechanism of sex determination, suggesting that males are heterogametic (XY) and females homogametic (XX) (John 1957). However, given advancements in cytogenetic methodologies and knowledge, these findings are now considered questionable and warrant reevaluation. To address this, we applied classical and advanced molecular cytogenetic techniques, including FISH mapping of 18S and 5S ribosomal genes and histone H3 genes. These approaches aimed to provide new insights into the karyotypic organization of *A.ranae* and to facilitate comparative analyses with other species within the order Echinorhynchida.

## ﻿Materials and methods

### ﻿Frog sampling and parasite specimen collection

Frog hosts of *Acanthocephalusranae* were collected from two locations in May and June 2023: *Pelophylaxridibundus* specimens were captured in Veľký Lel (47°45'35.4"N, 17°56'41.0"E), and *P.esculentus* in Rusovce (48°03'26.8"N, 17°09'11.2"E). A total of ten specimens from each species were sampled under a permit issued by the Ministry of Environment of the Slovak Republic (No. 519/2022-6.3), with all procedures approved by the Ethics Committee of the Institute of Parasitology, Slovak Academy of Sciences. Frogs were euthanized using a clove oil-water emulsion (20 drops of clove oil [Sigma-Aldrich] per liter of water) for anesthesia, followed by spinal cord severance. Specimens were either processed on-site or transported to the laboratory for dissection. From the frog intestines, 38 *A.ranae* parasites were isolated, rinsed in 0.9% saline, and microscopically identified as 21 males and 17 females. The samples were then divided into two groups: one was immediately processed for cytogenetic analysis, while the other was fixed in 100% ethanol and stored at –20 °C for subsequent genomic DNA extraction.

### ﻿Cytogenetic analysis

Live specimens of *A.ranae* were incubated at room temperature (RT) in a 0.025% colchicine solution for one hour. Parasites were then subjected to hypotonic treatment in 0.075 M KCl at RT. Whole, intact females were incubated in the hypotonic solution for 4–5 h, whereas only the testes of males were incubated for 20 min. Fixation was performed in two steps using freshly prepared modified Carnoy’s fixative (methanol:acetic acid, 3:1) for 30 min and 15 min. Fixed samples were stored at –20 °C until further analysis. Chromosome slides were prepared following the method described in Orosová and Špakulová (2018). Slides of sufficient quality were dehydrated in a graded ethanol series (70%, 80%, and 100%; 1 min each), air-dried at RT, and stored at –20 °C. Classical cytogenetic analysis was performed on Giemsa-stained slides (5%, pH 6.8, 30 minutes, RT). Basic chromosome characteristics, including number, shape, and size, were determined from digital images of ten high-quality mitotic metaphases from both sexes at 100 × magnification. Absolute length, relative length, and centromeric index were calculated (details in Orosová et al. 2022), with the mean and standard deviation of each chromosome pair and its arms calculated using Microsoft Excel. Chromosomes were classified following Dos Santos Guerra (1986). In addition, CG- and AT-rich heterochromatin regions were labeled using chromomycin A_3_ (CMA_3_) and 6-diamidino-2-phenylindole (DAPI), respectively, according to the protocol described in Orosová et al. (2023).

### ﻿DNA extraction and DNA probes preparation

Genomic DNA was extracted using the QIAamp® DNA Kit (QIAGEN, Hilden, Germany) following the manufacturer’s instructions. The extracted gDNA was used for both sequencing and polymerase chain reaction (PCR).

The 18S rDNA probe was prepared by PCR with specific primers, Acant18SF (5′-AGATTAAGCCATGCATGCGTAAG-3′) and Acant18SR (5′-TGATCCTTCTGCAGGTTCACCTAC-3′) (Perrot-Minnot 2004). The PCR conditions were as follows: initial denaturation at 95 °C for 3 min, followed by 30 cycles of 94 °C for 1 min, 60 °C for 30 s, 72 °C for 90 s, and a final extension at 72 °C for 10 min. The size of the PCR product was verified on a 1% agarose gel in TAE buffer, and the product was subsequently sequenced using Sanger sequencing at SEQme (Dobříš, Czech Republic). The sequencing yielded a 1627 bp long sequence, which has been deposited in GenBank under accession number PQ277049. The 18S rDNA probe was labeled with digoxigenin-11-dUTP (Roche Diagnostics, Mannheim, Germany) according to the nick translation protocol described in Hejníčková et al. (2021), with a reaction time of 45 min at 15 °C.

The 5S rDNA fragment was amplified using primers specific for acanthocephalans, Acanth5SF (5′-GTGATCGAACGAGAACCGGT-3′) and Acanth5SR (5′-TCACAAACTTTCGCGCGTTA-3′) (Orosová and Marková 2024). The PCR cycle conditions were: initial denaturation at 95 °C for 3 min, followed by 35 cycles of 94 °C for 30 s, 59 °C for 30 s, and 72 °C for 90 s, with a final extension at 72 °C for 3 min. The H3 histone gene fragment was amplified by PCR from *A.ranae* gDNA using the degenerate primers H3aF (5′-ATGGCTCGTACCAAGCAGAC(ACG)GC-3′) and H3aR (5′-ATATCCTT(AG)GGCAT(AG)AT(AG)GTG AC-3′) (Cabrero et al. 2009). PCR products of the 5S rDNA and H3 histone genes were run on a 1% agarose gel to verify amplification and fragment sizes. Both PCR products were purified using the Wizard SV Gel and PCR Clean-Up System (Promega) according to the manufacturer’s instructions and subsequently sequenced via Sanger sequencing at SEQme (Dobříš, Czech Republic) and confirmed as the H3 histone gene and 5S rDNA by BLAST search. For the newly obtained histone H3 gene sequence of *A.ranae*, species-specific primers ARH3F (5′-CAGCCAGAAAGACAGCGTTG-3′) and ARH3R (5′-GGAATCGCAGGTCCGTTTTG-3′) were designed using Geneious Prime v. 2021.1.1 software. Using this primer set, a 183 bp fragment of the H3 gene was amplified under the following PCR conditions: 94 °C for 30 s, 59 °C for 30 s, 72 °C for 60 s, for 35 cycles. The amplified 5S rDNA and H3 fragments were labelled by PCR with dNTP mix containing 0.35 mM biotin-16-dUTP (Roche Diagnostics).

### ﻿Fluorescence in situ hybridization (FISH)

The labeled probes were used in single- or two-color FISH according to the protocol described in Cabral-de-Mello and Marec (2021). For both FISH procedures, the hybridization solution for each slide contained 50% of deionized formamide (v/v), 20% of dextran sulfate (v/v), 10% of 2 × SSC, and approximately 50–100 ng of each labeled probe. The total volume of hybridization solution, including probe, was 20 µl for single- and 30 µl for two-color FISH. Probe denaturation was secured by incubating the solution at 95 °C for 10 minutes, followed by immediate cooling on ice for 3–5 min. The denatured probes were subsequently applied to the slides, covered with a glass coverslip and incubate at 68 °C for 3 min and 30 s. After this, the slides were placed in a humid chamber and incubated overnight at 37 °C. Following stringent washes, 2 × SSC, 0.1 × SSC, and WBB (washing blocking buffer, 4 × SSC, 0.1% v/v Tween 20, 1% w/v skimmed milk) were performed at RT. The immunological detection of biotinylated probes was conducted using the Cy3-conjugated streptavidin (Jackson ImmunoResearch Europe Ltd., Cambridgeshire Business Park, Ely, UK) amplified with biotinylated anti-streptavidin (Vector Labs. Inc., Burlingame, CA, USA) and re-detected with Cy3-conjugated streptavidin. The detection of DIG-labeled probes was performed using Alexa Fluor® 488-Monoclonal Mouse Anti-Digoxin Antibody, followed by amplification with Alexa Fluor® 488-F(ab’)2 Goat Anti-Mouse IgG (H+L) (min X) Secondary Antibody (both Jackson ImmunoResearch Europe Ltd.). Finally, the slides were counterstained with DAPI in ProLong Antifade medium (Invitrogen, Carlsbad, CA, USA), covered with a glass coverslip, sealed with nail polish, and stored in dark at 4 °C until further use. Stained slides were investigated using a LEICA DM 4000 B combined light and fluorescence microscope equipped with a DFC 450 C digital camera. Separately captured images for each fluorescent dye were pseudocolored and merged using Adobe Photoshop version 7.0.

## ﻿Results

### ﻿Karyotype characteristics and course of meiosis

**Figure 1.**
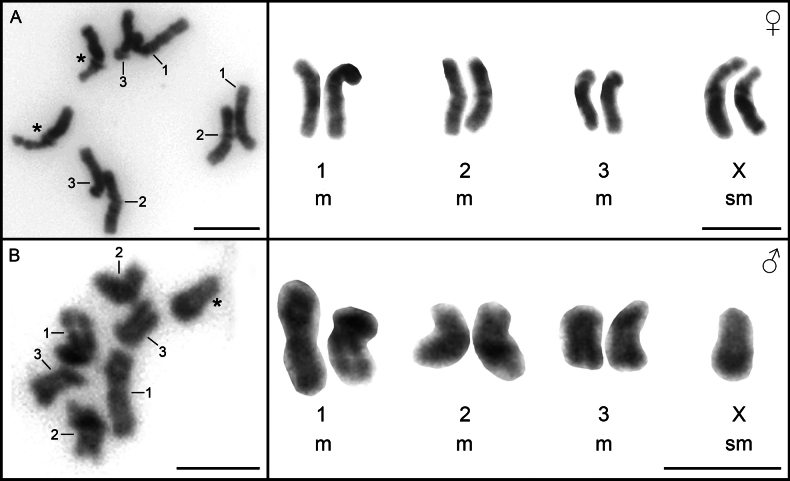
Mitotic metaphases (left panel) and assembled karyotype (right panel) of *Acanthocephalusranae*. **A.** Female chromosome spread with noticeable secondary constriction on X chromosomes; **B.** Male chromosome spread. Asterisks indicate X chromosomes. Scale bars: 5 µm.

The diploid number 2n = 7/8 was found in all cells examined and the chromosome complement consisted of three pairs of autosomes and two X chromosomes in the females (Fig. 1A) and one X chromosome in the males (Fig. 1B). The karyotype consists exclusively of bi-armed chromosomes, whereby the autosomes are metacentric (m) and the sex chromosomes are submetacentric (sm) and the karyotype formula is 2n = 7/8; n = 3m + 1sm (X). Homologous chromosomes identification is quite difficult, but the sex chromosomes have a visible secondary constriction on the short arms (Fig. 1A), which indicates the presence of the nucleolus organizer region (NOR) and allows easy identification of the sex chromosomes. The chromosomes are relatively small, ranging in size from 5.18 µm to 3.98 µm. The summary of the morphometric analysis obtained from the measurement of 10 Giemsa-stained metaphase plates can be found in Table 1.

**Table 1.** Measurement (mean ± SD) and classification of chromosomes of *Acanthocephalusranae*.

Chromosome number	Absolute length (mean ± SD^a^) (μm)	Relative length (mean ± SD) (%)	Centromeric index (mean ± SD)	Classification^b^
1	5.18 ± 0.33	29.38 ± 1.86	47.35 ± 0.85	m
2	4.55 ± 0.23	25.80 ± 1.33	47.30 ± 1.28	m
3	3.93 ± 0.18	22.24 ± 1.04	49.04 ± 0.79	m
X	3.98 ± 0.27	22.57 ± 1.52	35.85 ± 2.75	sm

^a^ Mean absolute lengths were calculated from 10 best mitotic metaphases of *A.ranae*. ^b^ Classification according to Dos Santos Guerra (1986), m – metacentric, sm – submetacentric chromosome pair.

**Figure 2.**
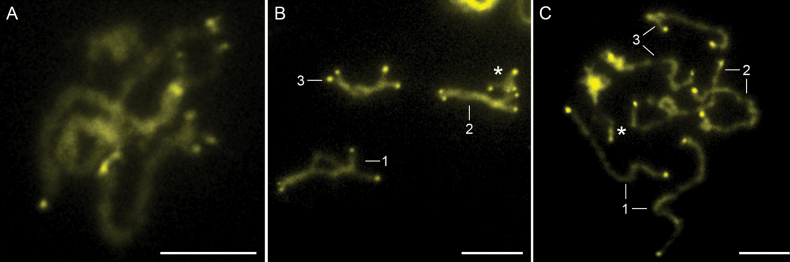
Chromosomes of *Acanthocephalusranae* stained with chromomycin A_3_ indicating GC-rich heterochormatin. **A.** Pachytene nucleus; **B.** Male diplotene nucleus; **C.** Anaphase II. Asterisks indicate X chromosomes. Scale bars: 5 µm.

Differential staining with 4’,6-diamidino-2-phenylindole (DAPI) and chromomycin A_3_ (CMA_3_) showed a very low presence of AT-rich heterochromatin (DAPI^+^) on the chromosomes of *A.ranae*, mainly centromeric heterochromatin, whereas bright bands of GC-rich heterochromatin (CMA_3_^+^) were detected at the ends of all chromosomes (Fig. 2). These fluorescent bands were most pronounced in the pachytene and diplotene stages of the nuclei (Fig. 2A, B).

The meiosis of *A.ranae* (Suppl. material 1) followed the expected pattern, beginning with leptotene, during which the chromatin formed long, thin strands. This progressed into zygotene, where bivalents began to form (Suppl. material 1: fig. S1B). During pachytene and diplotene, females had four bivalents (Suppl. material 1: fig. S1C), while males had three bivalents and one univalent (Suppl. material 1: fig. S1D). The division proceeded via metaphase I (Suppl. material 1: fig. S1E, F) to the second meiotic division and via metaphase II (Suppl. material 1: fig. S1G) to anaphase II, in which the chromosomes separated into chromatids (Suppl. material 1: fig. S1H), leading to the formation of haploid gametes.

### Distribution of ribosomal RNA and histone H3 genes

**Figure 3.**
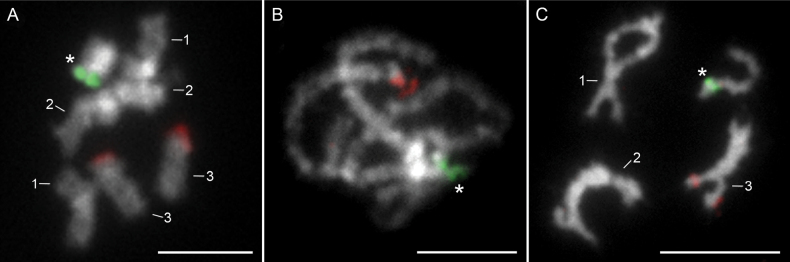
Two-color FISH with 18S (green) and 5S (red) rDNA probes on chromosomes of *Acanthocephalusranae*. **A.** Male metaphase nucleus; **B.** Pachytene nucleus; **C.** Male diplotene. Asterisks indicate X chromosomes. Chromosomes were counterstained with DAPI. Scale bars: 5 µm.

Two-color FISH experiments with probes of both rDNA families, 18S and 5S rDNA, showed a distinct and separate cluster distribution of each marker on the chromosomes of *A.ranae* (Fig. 3). Single cluster per haploid genome were observed for both major and minor rDNA. The major ribosomal genes were located on the short arms of the sex chromosomes at the site of secondary constriction (Fig. 3, green signal). In contrast, the 5S rDNA showed a hybridization signal on the third pair of autosomal chromosomes at a distal (telomeric) site (Fig. 3, red signal). The sequencing of the 5S rDNA of *A.ranae* resulted in a sequence length of 197 bp. Comparison with the 5S rDNA sequences of *A.lucii* and *A.anguillae* (Orosová and Marková 2024), revealed a highly conserved 131 bp coding region showing 100% identity. The sequence has been deposited in GenBank under accession number PQ285389.

PCR generation of the specific histone H3 fragment resulted in a single band of the expected size (Suppl. material 2: fig. S2A). The PCR product was successfully labeled with biotin-16-dUTP and subsequently used as a probe for FISH (Suppl. material 2: fig. S2A). Although we used different probe mixtures with up to 100 ng of biotin-labeled probe and a sandwich-like system for signal amplification, we could not localize the hybridization signal precisely and found only a few spots on and near individual chromosomes with no discernible pattern (Suppl. material 2: fig. S2B–D). Negative results from FISH experiments indicate that the genomic arrangement of this gene is not suitable for FISH mapping. The reasons for these negative results are discussed below.

## ﻿Discussion

The earliest study on the chromosome number of *A.ranae* dates back to 1891, when Hamann (1891) identified it as 2n = 16, although details on chromosome morphology and sex determination were lacking. John (1957) later revised the number to 2n = 8 and found that all chromosomes were metacentric. He also observed two different karyotypes: females had four identical pairs of chromosomes, while males had three identical pairs and one pair of differing chromosomes, suggesting a heteromorphic XX/XY sex determination system. Our study used modern molecular karyology techniques and showed that females possess three pairs of autosomes and two identical sex chromosomes, while males have three pairs of autosomes and only one sex chromosome, resulting in a diploid number of 2n = 7 in males and 2n = 8 in females. No Y chromosome was detected in the male samples. The morphology of the autosomes, which are all metacentric, is consistent with the work of John (1957), but differences in the morphology of the X chromosome were observed, which we identified as submetacentric, in contrast to the metacentric X chromosome reported by John. Given that the previous study on the chromosomes of *A.ranae* was conducted over 65 years ago, and considering the significant advancements in laboratory techniques and equipment since then, it is probable that the observed differences in sex determination mechanisms and chromosome morphology are attributable to variations in counting and measurement methods. These discrepancies likely stem from the limitations of chromosome slide preparation and optical technology available at that time. Nonetheless, given the distinct host genera and the considerable geographic separation between the populations studied (Slovakia and Cardiff, Wales), the observed variation may reflect the presence of two separate species of acanthocephalans. Alternatively, inter-population variability cannot be ruled out.

Of the 470 Acanthocephala species assigned to the order Echinorhynchida, karyotypic analyzes were performed for only nine species, representing less than 2% of the total (see Orosová et al. 2023: suppl. table S1). These studies have consistently revealed a low and stable diploid chromosome number of 2n = 7/8, which was proposed by Orosová et al. (2023) as a characteristic karyological feature of the order. An outlier to this pattern is *Leptorhynchoidesthecatus*, which has a diploid number of 2n = 5/6 (Bone 1974). In contrast, the closely related species *L.plagicephalus* conforms to the typical 2n = 7/8 configuration (Fontana et al. 1993). This discrepancy could indicate chromosomal variations within the genus or necessitate a karyological re-evaluation of *L.thecatus*, as no detailed information on chromosomal morphology is currently available.

*Acanthocephalusranae* shares its diploid chromosome number (2n = 7/8) with other congeners, *A.anguillae* and *A.lucii* (Mutafová et al. 1997; Špakulová et al. 2002; Orosová et al. 2023; Marková et al. 2024). In *A.ranae*, all autosomes are metacentric, whereas *A.anguillae* and *A.lucii* display greater chromosomal diversity (Orosová et al. 2023; Marková et al. 2024). The sex chromosomes in *A.ranae* are submetacentric, contrasting with the acrocentric sex chromosomes of the other species. A shared karyotype feature of all three karyotypes is the metacentric pair No. 1 and the bi-armed, meta- or submetacentric morphology of chromosome pair No. 2. However, pronounced differences in the morphology are evident in chromosome pair No. 3, which is acrocentric in *A.anguillae* (Orosová et al. 2023), submetacentric in *A.lucii* (Špakulová et al. 2002; Marková et al. 2024), and metacentric in *A.ranae* (this study). These morphological variations suggest that intrachromosomal rearrangements, pericentromeric inversions, contributed to speciation of these species by altering chromosome morphology (shifting of the centromere position) without changing chromosome number. Inversions are known to play a crucial role in adaptation by reducing recombination between advantageous allele combinations (Kirkpatrick and Barton 2006; Lowry and Willis 2010). Furthermore, they are also important in speciation processes as they prevent recombination between locally adapted alleles and those that contribute to assortative mating (Trickett and Butlin 1994). Physical mapping of various cytogenetic markers improves the study of chromosomal structure and karyotypic evolution and has the potential to advance phylogenetic studies, as the spatial distribution of markers can provide crucial insights into chromosomal rearrangements during evolution (Yoshido et al. 2005; Nguyen et al. 2010). In this study, the ribosomal RNA genes (18S and 5S rDNA) on the chromosomes of *A.ranae* were mapped for the first time. Given their dynamic evolution, rRNA genes are valuable markers for the study of chromosomal evolution, both between closely related species and within individual species. Major ribosomal RNA genes (18S, 5.8S, and 28S) are typically arranged in tandem clusters with hundreds to thousands of copies (Prokopowich et al. 2003). In contrast, 5S rRNA genes exhibit more variable distribution patterns and form clusters with dozens to thousands of copies (Cabral-de-Mello et al. 2010) or occur as single copies scattered across the genome (Vierna et al. 2013). Within the order Echinorhynchida, the chromosomal distribution of rDNA has so far been mapped in five species (with *A.ranae*) using FISH (Bombarová et al. 2007; Orosová et al. 2023; Marková et al. 2024) and the apparent variability of location on the chromosome is evident. The Pomphorhynchidae, *Pomphorhynchuslaevis* and *P.tereticollis* have the first two NOR-bearing chromosome pairs with two rDNA clusters per haploid genome (Bombarová et al. 2007). The clusters on the second pair of chromosomes differ in the positioning between the two species, a feature that has been suggested to contribute to the diversification of these closely related and morphologically similar species (Bombarová et al. 2007). In Echinorhynchidae, three species of the genus *Acanthocephalus* differ in the organization of rDNA clusters. *A.lucii* and *A.ranae* each have a single rDNA cluster per haploid genome located on the short arms of the X chromosome (Marková et al. 2024; present work), whereas *A.anguillae* has three rDNA clusters per haploid genome, distributed on the first two pairs of autosomes (Orosová et al. 2023). Based on the results of molecular phylogenetic studies, *A.ranae* and *A.lucii* are more closely related to each other, forming a separate branch (Amin et al. 2019; Chaudhary et al. 2020; Lisitsyna et al. 2023; Zhao et al. 2023), than to *A.anguillae*. This has support also in our results. Moreover, the interstitial placement of the 18S loci on the first two autosomes in *A.anguillae* makes it more similar to species of the family Pomphorhynchidae than to its closely related species in the genus *Acanthocephalus*. The number and position of rDNA loci can change dynamically (Nguyen et al. 2010; Ferretti et al. 2019; Provazníková et al. 2021), and the available, albeit limited, data indicate the high mobility of 18S rDNA in acanthocephalans and its modification by different mechanisms. However, the similar morphology of the first two pairs of chromosomes, which house rDNA genes in *A.anguillae*, suggests they were not affected by large-scale chromosomal rearrangements. Additionally, the increased number of rDNA clusters in *A.anguillae* is not associated with an increase in chromosome number, as all three *Acanthocephalus* species share the same stable diploid chromosome number (2n = 7/8). Changes in the chromosomal positions of NORs have been reported in both animal and plant species, together with evidence for gain or loss of rDNA loci during evolution (Roy et al. 2005; Pérez-García et al. 2014; Rosato et al. 2015; Yucel et al. 2022). For some insect species, the association of rDNA and the sex chromosome has even been proposed as an ancestral character (Drosopoulou et al. 2012). The loss of X-linked NOR and the acquisition of an NOR on the first and second chromosomes could have occurred in the ancestral lineage before the separation of two branches, one forming by *A.ranae* and *A.lucii* and second with *A.anguillae*, *A.dirus* and *A.nanus* (see Fig. 1 in Chaudhary et al. 2020). However, whether sex chromosome location and single rDNA cluster (*A.lucii*, *A.ranae*) represent the ancestral status, with interstitial rDNA loci (*A.anguillae*) representing a derived pattern of distribution, or the opposite, remains uncertain. We can hypothesize that the increase or reduction in rDNA loci from the ancestral acanthocephalan karyotype with 2n = 7/8 may result from transposon activity and amplification or deletion through ectopic recombination. To answer the direction of karyotype evolution, studies on other species from different Echinorhynchida families still need to be carried out. However, we propose that the two interstitial rDNA clusters observed within a single bivalent in *A.anguillae* represent a derived pattern of rDNA distribution. This arrangement may have originated through intrachromosomal rearrangements, such as inversions, or by the transposition of a small number of rRNA genes to a new chromosomal locus, followed by their amplification via unequal crossing-over. Both interstitial clusters are colocalized with strong heterochromatin blocks, which are rich in repetitive sequences and could facilitate their spread. Additional support for this hypothesis comes from the considerably smaller size of one of the two interstitial clusters on bivalent No. 2 in *A.anguillae*, which may represent an evolutionarily recent rDNA site.

A common pattern of 5S rDNA distribution on chromosomes is evident in three *Acanthocephalus* species (Orosová and Marková 2024; this study), where FISH analysis revealed a single cluster located at a subtelomeric site on the short arms of chromosome pair No. 3. The difference lies in the morphology of this pair of chromosomes, which is metacentric in *A.ranae*, submetacentric in *A.lucii* and acrocentric in *A.anguillae* (Orosová and Marková 2024; this study). The “inversion” hypothesis was proposed for the change in chromosome morphology, but not chromosome number, with the 5S rDNA cluster remaining in the same location in *A.lucii* and *A.anguillae* (see schematic drawing in Fig. 2 in Orosová and Marková 2024). The same applies also for *A.ranae* chromosome. The uniform pattern of 5S distribution may indicate that one subtelomeric cluster is an ancestral character of the family Echinorhynchidae. Independent signals of minor and major rDNA clusters located on different chromosome pairs were found in all three species. The observed spatial separation of ribosomal genes (18S and 5S rDNA) is characteristic of animal karyotypes, which typically exhibit greater separation of rDNAs compared to plants. According to the animal rDNA database, approximately 75% of animal karyotypes have 5S and 45S loci on separate chromosomes, while only 25% have co-localization (Sochorová et al. 2018).

Histone genes are well-known for their protein sequence conservation and conserved genomic distribution (Zhang et al. 2007; Silva et al. 2013), making them an ideal marker since they truly reflect the chromosomal rearrangements (Zhang et al. 2007). Previously, one interstitial histone H3 gene cluster near the centromere on the long arms of chromosome pair No. 1 was observed in *A.lucii* (Marková et al. 2024) whereas multiple loci unevenly distributed on all (including Bs) chromosomes were detected in *A.anguillae* (Orosová et al. 2023). In our analysis, PCR amplification and probe labeling of both degenerate and specific H3 PCR products proceeded as expected, resulting in the labeled probe of correct size. However, the FISH resulted in scattered hybridization signal across all chromosomes, without any distinct pattern. Additionally, some signals appeared off-target, possibly representing nonspecific fluorescent artifacts. BLAST analysis of the H3 sequence generated with the degenerate primers identified it primarily as histone H3, but further translation into amino acids and subsequent BLAST searches revealed similarity histone H3.3 of *Hofsteniamiamia* (93.81%). This suggests that the degenerate primers may have amplified histone H3.3 variant genes. Unlike canonical histone H3 genes, which are typically part of large, clustered gene families organized in repetitive arrays on chromosomes, H3.3 variants are often found in transcriptionally active regions (euchromatin) across the genome (Goldberg et al. 2010; Shi et al. 2017; Sokolova et al. 2023), which are below the standard resolution limits of FISH technique. Given the successful PCR amplification and fluorescent labeling, this interpretation appears to be the most plausible explanation for the observed non-specific mapping pattern on *A.ranae* chromosomes.

Differential staining with chromomycin A_3_ revealed GC-rich heterochromatin at chromosome ends of all chromosomes in *A.ranae*, just like in *A.lucii* and *A.anguillae* (Orosová et al. 2023; Marková et al. 2024). While the telomeric motif in acanthocephalans remains unknown, studies suggest it may involve a novel GC-rich sequence or a unique telomere maintenance mechanism (Bombarová et al. 2009; Orosová et al. 2023; Marková et al. 2024). To unravel this sequence comprehensive analyses, including genome sequencing and the application of bioinformatics tools for repeat identification and characterization, are essential.

## ﻿Conclusions

This study used molecular cytogenetics and differential staining to analyze the karyotype of *A.ranae*, contributing to the understanding of Acanthocephala chromosome structures. Inversion appears to be typical chromosomal rearrangements of Echinorhynchida species, as its important role in altering chromosome morphology has been demonstrated for all species studied to date. The mapping of 5S and 18S rDNA confirmed their clustered organization, emphasizing their potential as markers for comparative cytogenetic analysis. As mentioned above, the 5S rRNA genes were conserved in both number and chromosomal location, whereas mapping of 18S rDNA showed rather dynamic evolution. The histone H3 genes exhibited variability in their organization: no clear hybridization signals were detected in *A.ranae*, while scattered signals appeared across all chromosomes in *A.anguillae*. Conversely, *A.lucii* displayed a distinct organization with H3 genes localized in a single cluster. Further studies should focus on a deeper molecular analysis of histone H3 sequences, as the genus *Acanthocephalus* offers intriguing material for such research. More comprehensive comparative cytogenetic studies, including the evaluation of cytogenetic markers, could contribute to the clarification of unresolved phylogenetic and taxonomic questions in acanthocephalans.

## ﻿Additional information

### Author ORCIDs

Martina Orosová https://orcid.org/0000-0001-8934-9126

Anna Marková https://orcid.org/0000-0002-0824-2026

### Data availability

Sequence data that support the findings of this study have been deposited in the European Nucleotide Archive with the primary accession code PQ277049 and PQ285389.

## Supplementary materials

Supplementary material 1 Meiotic division of *Acanthocephalusranae* Data type tif https://binary.pensoft.net/file/1360950This dataset is made available under the Open Database License (http://opendatacommons.org/licenses/odbl/1.0/). The Open Database License (ODbL) is a license agreement intended to allow users to freely share, modify, and use this Dataset while maintaining this same freedom for others, provided that the original source and author(s) are credited.Martina Orosová, Anna Marková

Supplementary material 2 PCR generation of histone H3 probe and fluorescent mapping onto chromosomes of *Acanthocephalusranae* Data type tif https://binary.pensoft.net/file/1360951This dataset is made available under the Open Database License (http://opendatacommons.org/licenses/odbl/1.0/). The Open Database License (ODbL) is a license agreement intended to allow users to freely share, modify, and use this Dataset while maintaining this same freedom for others, provided that the original source and author(s) are credited.Martina Orosová, Anna Marková
