# Supplementary figures and images for: ﻿Karyological analysis of Acanthocephalusranae (Echinorhynchida): expanding the cytogenetic knowledge in acanthocephalans
Source: Zookeys. 2025 Jun 25;1243:173–89. doi: 10.3897/zookeys.1243.153591 (PMC12239011; doi:10.3897/zookeys.1243.153591)

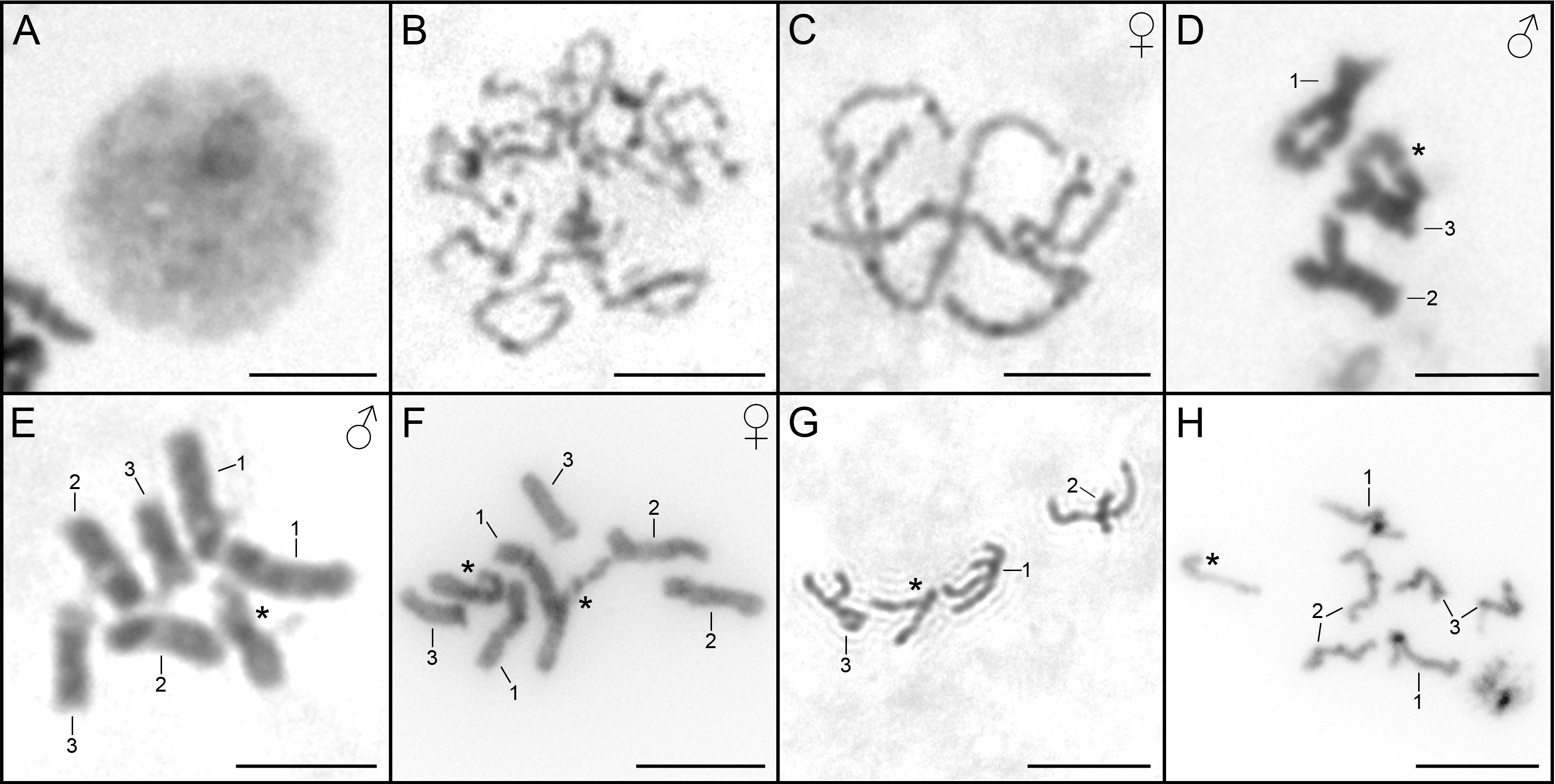

Supplement: Supplementary material 1 — Meiotic division of Acanthocephalusranae [file zookeys-1243-173_article-153591__-s001.tif]

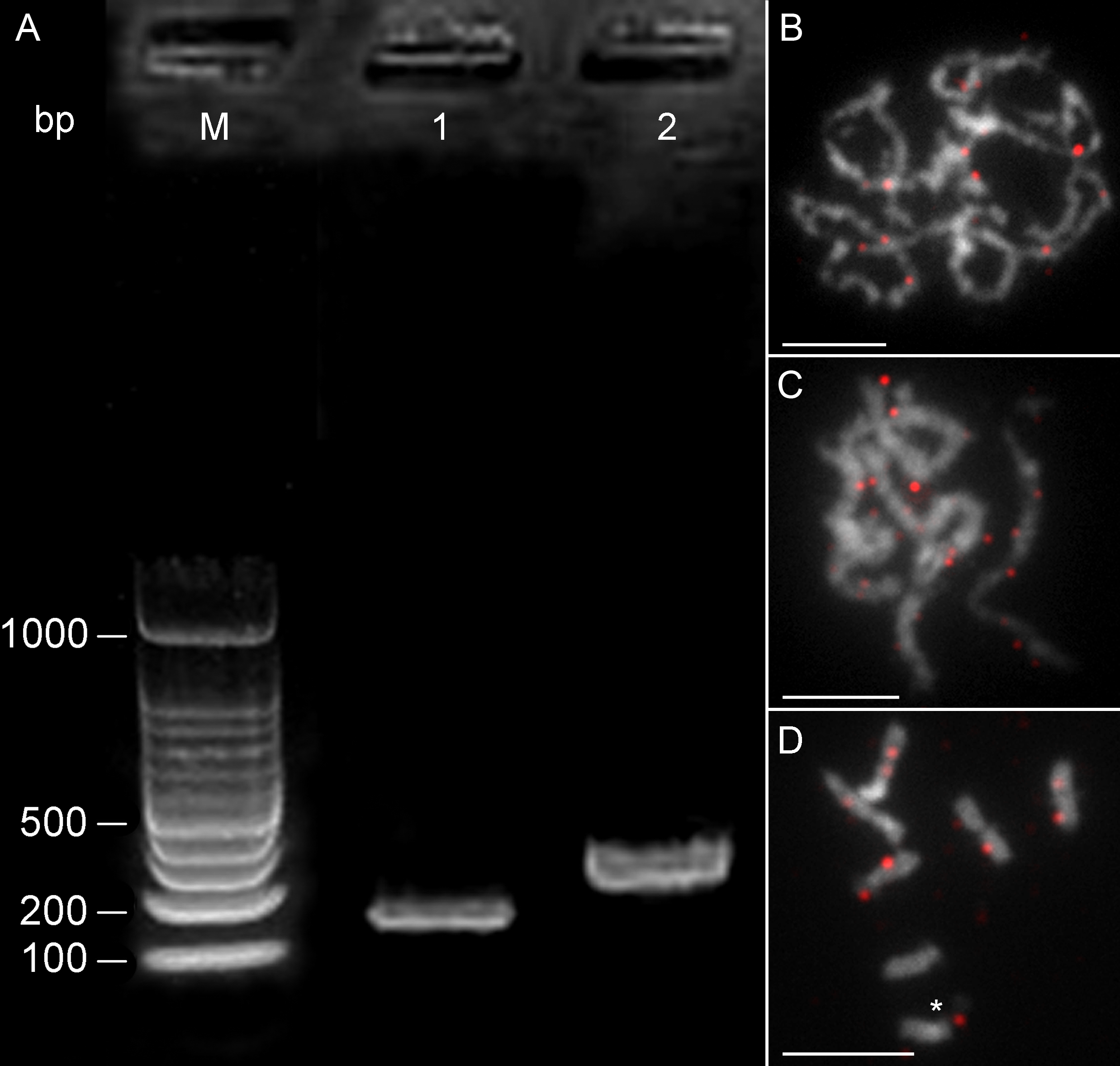

Supplement: Supplementary material 2 — PCR generation of histone H3 probe and fluorescent mapping onto chromosomes of Acanthocephalusranae [file zookeys-1243-173_article-153591__-s002.tif]
